# Compounds Derived from the Bhutanese Daisy, *Ajania nubigena*, Demonstrate Dual Anthelmintic Activity against *Schistosoma mansoni* and *Trichuris muris*

**DOI:** 10.1371/journal.pntd.0004908

**Published:** 2016-08-04

**Authors:** Phurpa Wangchuk, Mark S. Pearson, Paul R. Giacomin, Luke Becker, Javier Sotillo, Darren Pickering, Michael J. Smout, Alex Loukas

**Affiliations:** Centre for Biodiscovery and Molecular Development of Therapeutics, Queensland Tropical Health Alliance, Australian Institute of Tropical Health and Medicine, James Cook University, Cairns Campus, Cairns, Australia; Centers for Disease Control and Prevention, UNITED STATES

## Abstract

**Background:**

Whipworms and blood flukes combined infect almost one billion people in developing countries. Only a handful of anthelmintic drugs are currently available to treat these infections effectively; there is therefore an urgent need for new generations of anthelmintic compounds. Medicinal plants have presented as a viable source of new parasiticides. *Ajania nubigena*, the Bhutanese daisy, has been used in Bhutanese traditional medicine for treating various diseases and our previous studies revealed that small molecules from this plant have antimalarial properties. Encouraged by these findings, we screened four major compounds isolated from *A*. *nubigena* for their anthelmintic properties.

**Methodology/Principal Findings:**

Here we studied four major compounds derived from *A*. *nubigena* for their anthelmintic properties against the nematode whipworm *Trichuris muris* and the platyhelminth blood fluke *Schistosoma mansoni* using the xWORM assay technique. Of four compounds tested, two compounds—luteolin (**3**) and (3*R*,6*R*)-linalool oxide acetate (**1**)—showed dual anthelmintic activity against *S*. *mansoni* (IC_50_ range = 5.8–36.9 μg/mL) and *T*. *muris* (IC_50_ range = 9.7–20.4 μg/mL). Using scanning electron microscopy, we determined luteolin as the most efficacious compound against both parasites and additionally was found effective against the schistosomula, the infective stage of *S*. *mansoni* (IC_50_ = 13.3 μg/mL). Luteolin induced tegumental damage to *S*. *mansoni* and affected the cuticle, bacillary bands and bacillary glands of *T*. *muris*. Our *in vivo* assessment of luteolin (**3**) against *T*. *muris* infection at a single oral dosing of 100 mg/kg, despite being significantly (27.6%) better than the untreated control group, was markedly weaker than mebendazole (93.1%) in reducing the worm burden in mice.

**Conclusions/Significance:**

Among the four compounds tested, luteolin demonstrated the best broad-spectrum activity against two different helminths—*T*. *muris* and *S*. *mansoni*—and was effective against juvenile schistosomes, the stage that is refractory to the current gold standard drug, praziquantel. Medicinal chemistry optimisation including cytotoxicity analysis, analogue development and structure-activity relationship studies are warranted and could lead to the identification of more potent chemical entities for the control of parasitic helminths of humans and animals.

## Author Summary

Schistosomiasis and trichuriasis affects millions of people worldwide and are caused by blood flukes and whipworms, respectively. Only a handful of anthelmintic drugs exist to treat these infections and the pipeline for the next generation of anthelmintic drugs is sparse, precipitating the need for new drug development. In this context, medicinal plants present a viable source of novel anthelmintic compounds. This inspired us to study the selected naturally occurring compounds derived from a Bhutanese daisy medicinal plant, *Ajania nubigena* for their anthelmintic activities. Here, using the xWORM motility assay, we demonstrate that two compounds, luteolin (**3**) and (3*R*,6*R*)-linalool oxide acetate (**1**), display significant broad-spectrum anthelmintic activity against two of the most important genera of human helminth parasites, the nematode whipworm and the platyhelminth blood fluke. Luteolin exhibited the best activities with IC_50_ values of 5.8 μg/mL against schistosomes and 9.7 μg/mL against whipworms. Using scanning electron microscopy we showed that luteolin damages the tegument of blood flukes and induces abnormalities in the bacillary bands/glands and cuticles of whipworms. Intriguingly, our previous study showed that luteolin (**3**) was effective against multi-drug resistant *Plasmodium falciparum* malaria. Due to its broad-spectrum anti-parasitic activities, luteolin (**3**) is a desirable drug lead scaffold, which could be used for developing effective compounds to control and treat numerous tropical diseases.

## Introduction

The World Health Organization (WHO) recognises 17 different ‘neglected tropical diseases’ (NTDs) that affect more than 1.4 billion people in 149 countries [1]. Helminth infections caused by roundworms (nematodes) and flatworms (platyhelminths) comprise the largest group of NTDs [2]. Whipworms (Nematoda) cause trichuriasis and infect about 800 million people worldwide, second among the nematodes only to Ascaris infection [3]. The schistosome blood flukes (Platyhelminthes) cause schistosomiasis, a disease that afflicts more than 240 million individuals and kills hundreds of thousands each year [4].

A variety of approaches have been employed to combat these infections including education, vector control, sanitation and hygiene, behavioural change and mass drug administration (MDA) programs. Various *in vitro* and animal model studies have highlighted the repurposing of existing drugs and discovery and development efforts for new drugs [2, 5] but all things considered, the pipeline for the next generation of anthelmintic drugs is sparse. Indeed, a systematic assessment of databases of drug regulatory authorities and the WHO, as well as clinical trial registries, revealed that no new antiparasitic drugs have been approved during the last decade [6]. There are only a handful of anthelmintic drugs on the market, some of which have unwanted side effects or achieve poor cure rates due to primary drug resistance developing in the parasites [7–9].

For example, praziquantel, which is the sole frontline drug used in the mass treatment of schistosomiasis, is efficacious but has many disadvantages: a) it is ineffective against juvenile stages of the parasite, b) reduced efficacy has been reported in field studies [10], c) there is a strong possibility that praziquantel resistance could appear if sufficient selection pressure is applied and mass drug administration is continued [11], and d) its active (*S*)-enantiomer and inactive (*R*)-enantiomer components remain inseparable in the production process, rendering bulky tablets that discourages patients from taking the right doses or the complete dosing regimen, which could trigger the development of drug resistance [12]. Until new arsenals of safe and effective drugs and/or vaccines are made available, helminth infections will continue to affect the world’s most impoverished populations, causing significant morbidity and mortality worldwide.

While new drugs can be developed synthetically, natural products—especially the medicinal plants—have been an important pool of antiparasitic drugs. Quinine and artemisinin discovered from medicinal plants continue to save the lives of millions of people worldwide. As such, the notion of therapeutics derived from medicinal plants has re-surfaced [13]. Crude extracts and compounds of plant origin have been demonstrated to possess broad biological activities in *in vitro* and *ex vivo* assays and animal models of parasitic infections [14–19]. Edwards *et al*. [20] showed that 7-keto-sempervirol isolated from the boxthorn from which goji berries are harvested, *Lycium chinense*, was effective against *Schistosoma mansoni* and *Fasciola hepatica*. A compound that displays such broad anti-parasitic activity against various life stages of multiple parasites is highly desirable. Extracts of the Bhutanese medicinal plant from the flowering daisy family, *Ajania nubigena* (Syn. *Tanacetum nubigenum* DC.) have been previously shown to possess broad biological activities including antiparasitic effects against *Plasmodium falciparum* and antimicrobial properties [21]. It is locally known as *m*.*khan-d*.*kar* and has been used in Bhutanese traditional medicine (derived from Tibetan scholarly medicine) for thousands of years as incense and for treating an array of conditions and infections including wounds, bleeding and swelling [21]. Although this plant is not specifically indicated for treating intestinal worms, the decoction of its closely related species, *Tanacetum parthenium* L. (feverfew) and *Tanacetum dolichophyllum* Kitam has been traditionally used by the Ladakhis Amchis (medical system derived from Tibetan medicine and similar to Bhutanese traditional medicine) [22] and Costa Ricans healers [23] against intestinal worms. These plants have reserves of highly aromatic essentials oils that have evolved to aid in plant protection and competition against plant parasites and herbivorous insects. Chemically, these plants contain similar chemotypes including sesquiterpenes and flavonoids [23]. Encouraged by these lead information, we have investigated the anthelmintic properties of four compounds isolated from the Bhutanese *A*. *nubigena* against two of the most important genera of human helminth parasites, the nematode whipworm (*Trichuris*) and the platyhelminth blood fluke (*Schistosoma*). To monitor worm viability we used xWORM, a technique that monitors helminth motility in real time using xCELLigence [24–25]. The advantage of using xWORM over other methods is that it enables high-throughput screening of a large number of compounds in a fully automated, label-free manner.

## Materials and Methods

### Plant materials and preparation of compounds

**Fig 1:**
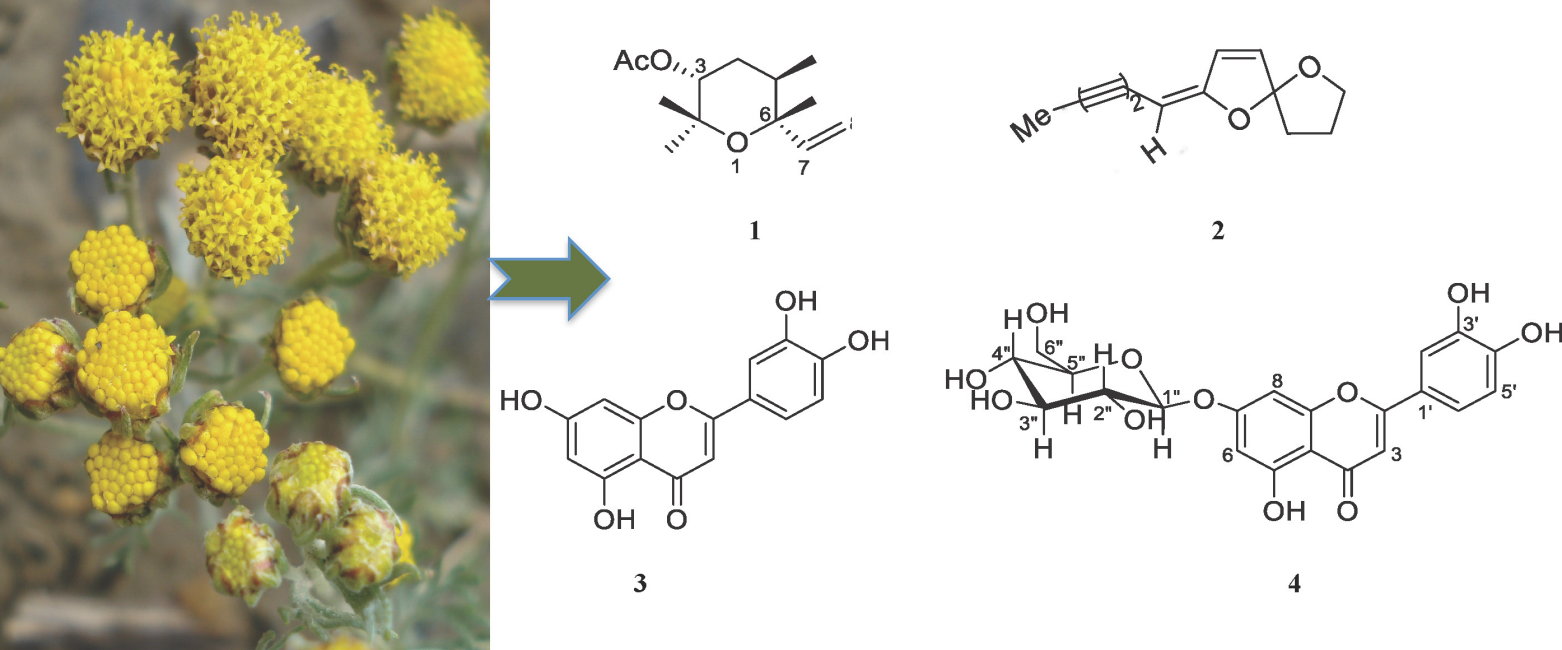
Structure of compounds [21]. (3*R*,6*R*)-linalool oxide acetate (**1**), (*E*)-spiroether (**2**), luteolin (**3**), luteolin-7-*O*-β-D-glucopyranoside (**4**) were derived from *A*. *nubigena* and tested for their anthelmintic activities using xWORM. Plant photo courtesy–PW.

The aerial part of wild *Ajania nubigena* was collected from alpine mountains (altitude range of 3600–4800 meters above sea level) of Lingzhi, Bhutan in August 2009. The collected plant material was air-dried and a herbarium specimen with voucher number 73 was deposited at the herbarium collection section of Menjong Sorig Pharmaceuticals, Ministry of Health, Bhutan. The air-dried plant material (2 kg) was chopped into small pieces and was repeatedly extracted with methanol (AR/HPLC grade, 3 L over 48 h). The extract was filtered and then concentrated using a Buchi rotary evaporator to generate a crude methanol (MeOH) extract (58.2 g). The isolation technique was described previously [21]. MeOH extract was dissolved in MeOH:H_2_O (200 mL, 1:9) and then fractionated with hexane followed by ethyl acetate to obtain the hexane extract (28.0 g) and the ethyl acetate extract (12.5 g), respectively. Subsequently, essential oil (EO) extraction was performed using hydro-distillation (60°C). One kg of dried plant material yielded 7 mL of pale green EO. The crude MeOH extract and EO were subjected to extensive natural products isolation processes. Flash column chromatography packed with Merck Kieselgel 60 PF254 and pre-coated silica plates (0.2 mm silica thickness, Merck) were used for repeated separation and purification of compounds. UV light (short wavelength of 254 nm, long wavelength of 366 nm) and ceric ammonium molybdate (CAM) were used for visualization and detection of compounds on Thin Layer Chromatography (TLC) plates. Eight compounds were isolated and characterised in total from the MeOH and EO extracts using Infrared (IR) Spectroscopy, Mass Spectrometry (ESI-MS, HR-EI-MS), Gas Chromatography Mass Spectrometry (GCMS), and Nuclear Magnetic Resonance (NMR-^1^H, ^13^C, gCOSY, gNOESY, TOCSY, gHSQC and gHMBC) [21]. In this study, we have selected four major compounds whose structures are produced in Fig 1: (3*R*,6*R*)-linalool oxide acetate (**1**), (*E*)-spiroether (**2**), luteolin (**3**) and luteolin-7-*O*-β-D-glucopyranoside (**4**).

The stock solutions of the four test compounds were prepared at the concentration of 100 mg/mL in DMSO and then subsequently diluted them with respective tissue culture media to make 10x solutions. 20 μl of 10× drug concentration was added to 180 μl of media containing helminths in the E-plate wells. Control worms were cultured in the presence of DMSO equivalent to that used for the highest drug concentration; this group was used to determine 100% motility. For schistosomula drug assays, stock solutions were diluted in culture media with two-fold dilutions with in-well concentrations of (2–1000 μg/mL).

### Preparation of *Schistosoma mansoni*

*S*. *mansoni* cercariae were shed from infected *Biomphalaria glabrata* snails (Biomedical Research Institute, MD, USA) by exposure to light at 26°C for 2 hours and used to infect 12–14 week old male BALB/c mice (120 cercariae/mouse) by abdominal penetration [26]. Adult flukes were perfused from the mesenteries 7 weeks post-infection and then transferred to Basch medium for culturing as previously reported [27]. To monitor the effects of drugs on *S*. *mansoni* schistosomula, shedding of cercariae from snails and subsequent *in vitro* transformation to schistosomula was performed as described by Peak et al [28].

### Preparation of *Trichuris muris*

Genetically susceptible mice (STAT6^*-1-*^) were orally infected with *T*. *muris* eggs (200 μL volume containing ~ 200 eggs) and sacrificed after 4 weeks. Adult worms were harvested from the caecum, washed with PBS/2x antibiotic/antimycotic (AA) and resuspended in 100 μl of RPMI containing 10% foetal calf serum and AA (culture medium) then transferred to E-plates for motility assessment using the xWORM assay.

### xWORM assay

The trematocidal effects of test compounds against *S*. *mansoni* were evaluated using an xCELLigence SP system (ACEA Biosciences) as described by us [25]. Adult flukes (1 fluke per well) were placed in triplicate for each compound into 96 well E-plates (ACEA Biosciences) containing 180 μl of culture medium and cultured overnight at 37°C with 5% CO_2_ to obtain a baseline motility reading. Test compounds were added to E-plates and motility was monitored for 12**–**40 hr. The inter-well spaces of E-plates were filled with 100 μL culture media. All experiments were carried out as per manufacturer’s instructions with 15 sec read intervals using the real time cell assay (RTCA) software (ACEA Biosciences) as described previously [24–25].

Similarly, the nematocidal effects of test compounds against *T*. *muris* were assessed using the same xCELLigence SP system as described above. We determined the optimal culture duration and worm concentration to maximize the signal to noise ratio using the xWORM technique for the first time with *T*. *muris*. Different numbers of adult *T*. *muris* (2, 4 and 8) were added to individual E-plate wells and motility was monitored overnight. Four worms of mixed gender per well in a final volume of 200 μl of culture medium was determined to be optimal for this study. The E-plates containing worms were treated with prepared concentrations of the test compounds and were monitored using the xCELLigence SP system for 12–40 hr. Inter-well spaces were filled with 100 μL of culture medium or PBS to prevent evaporation. Each set of conditions was monitored in triplicate.

### Determining the effects of compounds against schistosomula

The 96 well plates containing culture media were loaded with schistosomula (100 μL volume containing ~ 100 schistosomula) in triplicate and treated with the test compounds at various in-well concentrations of 2–1000 μg/mL. Plates were cultured at 37°C with 5% CO_2_ for 12–40 hr and were finally stained with trypan blue solution to assess final viability after treatment. The stained schistosomula were observed by light microscopy, and live and dead flukes in each well were counted manually and 50% inhibitory concentration (IC_50_) values were obtained.

### Determination of IC_50_ values for test compounds

The IC_50_ values of test compounds were determined based on the motility index for adult worms as described by us [25]. Briefly, motility index was calculated as the standard deviation (SD) over 800 data points (i.e. 4 readings per min for 200 min) of the cell index (CI) difference from the rolling average over 20 data points (10 proceeding and preceding CI values—5 min total). One hundred percent motility was determined from the average motility index of the untreated wells, while 0% motility was determined from a media only well (no worms present). The motility index averaged over 100 data points (25 min) was converted to percentage motility and this figure was used in GraphPad Prism 6.0 to calculate dose response curves. We used a log (test compound concentration) vs normalised response (100%–0%) formula, with variable slope when data were sufficient or set -1 hill slope when data was limited, and automatic removal of outliers (with default ROUT coefficient used: Q = 1.0%). IC_50_ values for each dose concentration were calculated at 1 hr, 6 hr, and 12 hr post-treatment of the worms with the test compounds. Compounds with IC_50_ values of higher than 100 μg/mL were considered ineffective in this study.

### Statistical analysis

Statistical analyses were undertaken using GraphPad Prism 6.0. When data were sufficient to use the variable slope analysis, the Hill Slope and the Log IC_50_ value were together compared for significant differences using an extra sum-of squares F-test. For the *in vivo* mouse experiments, 1-way ANOVA with Holm-Sidak’s multiple comparisons test was used for determining significance *p*-values.

### Scanning electron microscopy

Worms treated with the test compounds were prepared for scanning electron microcopy (SEM) as follows: a) fixed in 3% gluteraldehyde in Sorensen’s buffer overnight, b) dehydrated for 15 min in a graded ethanol series (50%, 60%, 70%, 80%, 90%, 100%), mixture of ethanol and hexamethyldisilizane (HMDS) (1:1 ratio) and then finally with pure HMDS (100%), c) the dehydrated worms were covered and left overnight in a fume hood to allow the HMDS to evaporate. Completely dried worms were placed on an aluminum stub (at least three worms from each treatment regimen), sputtered with gold and visualized using a JEOL JSM scanning electron microscope operating at 10 kV. Each worm on a stub was scanned from head to tail to determine if the compounds had altered its gross morphology. Digital image acquisition was performed on the affected region of the worms using Semaphore software.

### *In vivo* animal model studies of luteolin (3)

Four to five week-old STAT6^*-1-*^ mice were grouped (each group with 9 mice) as: solvent control, positive control and luteolin (**3**). Each mouse was orally infected with 200 μl of PBS containing approximately 200 live embyronated eggs of *T*. *muris*. These mice were housed for 4 weeks with constant access to water and pelleted food. After 4 weeks, luteolin (**3**) and the positive control drug (mebendazole) prepared in 1% DMSO/PBS were orally administered at a single dose of 100 mg/kg (9 mice in total for each group) as per the protocol [29–30]. Five days after one dose of oral treatment the mice were sacrificed, worms were harvested from the caecum, and counted manually using light microscopy. The recorded numbers of worms were averaged to find the percentage reduction in worm burden for each group of mice.

### Ethics and approvals

The permit to collect medicinal plants from the park management areas around Lingzhi, Bhutan was obtained from the Department of Forest, Ministry of Agriculture and Forestry in Bhutan. The material transfer agreement and approval was sought from the National Biodiversity Centre of Bhutan. MeOH extracts of the plants were transported to Australia with prior approval from the Bhutan Agriculture and Food Regulatory Authority, University of Wollongong and sample inspections by Australian Quarantine & Inspection Service in 2010. The James Cook University (JCU) animal ethics committee approved all experimental work involving animals (Ethic approval number A2213). Mice infected with *S*. *mansoni* and *T*. *muris* were raised in cages in the JCU animal facility for 4–7 weeks in compliance with the Australian Code of Practice for the Care and Use of Animals for Scientific Purposes, 7^th^ edition, 2007 and the Queensland Animal Care and Protection Act 2001. Mice were kept under normal conditions at regulated temperature (22°C) and lighting (12 hr light/dark cycle) with free access to pelleted food and water. All reasonable efforts were made to minimise the suffering of the mice.

## Results

The isolation and characterization of compounds from the *A*. *nubigena* plant were performed as reported previously by us [21]. Out of eight compounds isolated from this plant, several were reported to have antimalarial, antibacterial, antifungal and cytotoxicity activities [21]. Encouraged by the antimalarial activities, four major compounds (Fig 1): (3*R*,6*R*)-linalool oxide acetate (**1**), (*E*)-spiroether (**2**), luteolin (**3**) and luteolin-7-*O*-β-D-glucopyranoside (**4**), were tested for broad-spectrum anthelmintic activities against two distinct phyla of helminths, the platyhelminth trematode blood fluke *S*. *mansoni* and the nematode whipworm *T*. *muris*.

### Trematocidal activity of compounds against adult *S*. *mansoni*

**Fig 2:**
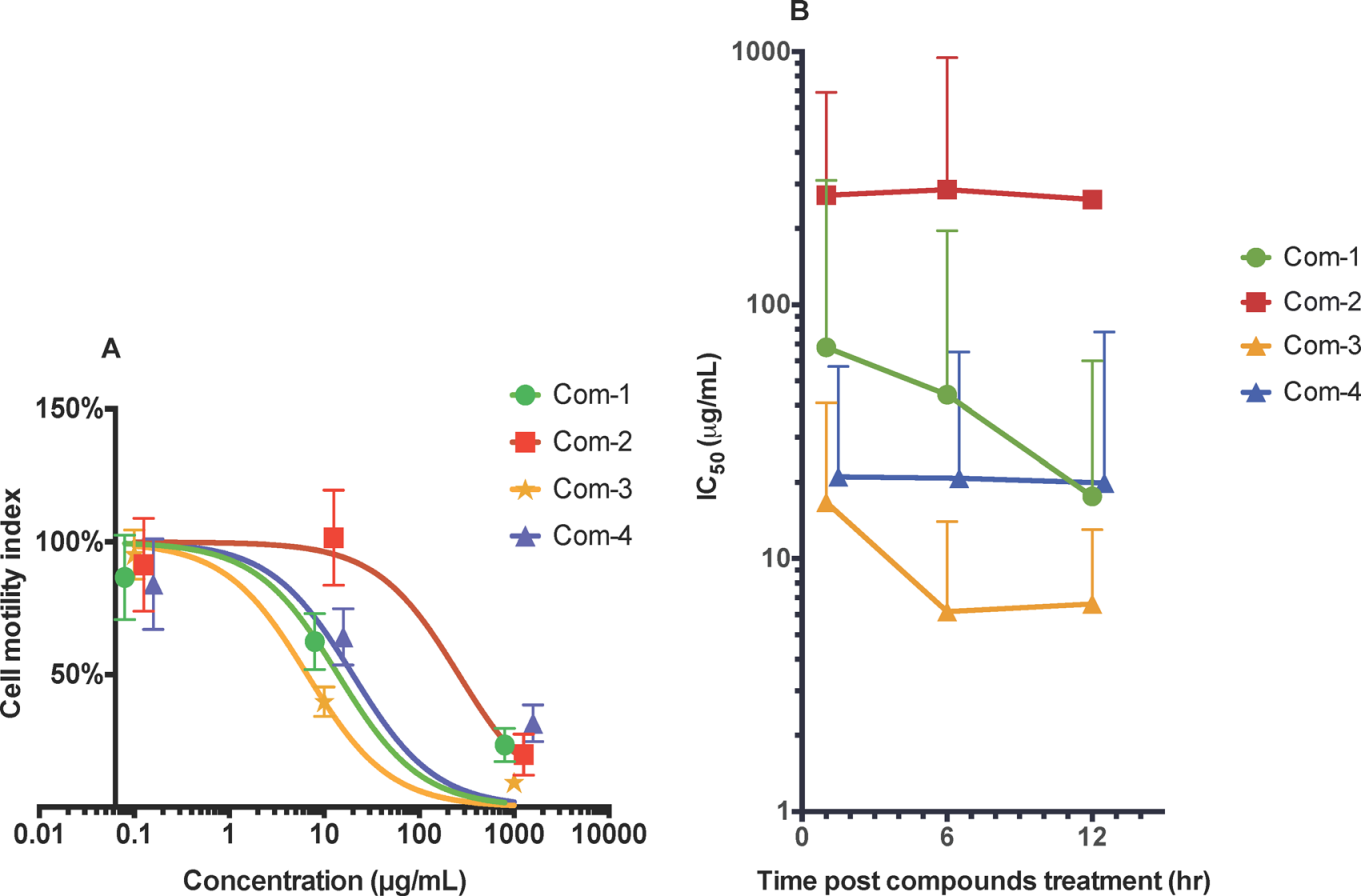
*In vitro* anti-schistosome activities of four compounds against adult *S*. *mansoni*. A) Motility index dose response curve of worms at the 12 hr time point when treated with (3*R*,6*R*)-linalool oxide acetate (**1**), (E)-spiroether (**2**), luteolin (**3**), luteolin-7-*O*-β-D-glucopyranoside (**4**) (abbreviated as Com-1, Com-2, Com-3 and Com-4) at different concentrations (0.1–1000 μg/mL). Motility index was calculated as the standard deviation (SD) over 800 data points (i.e. 4 readings per min for 200 min) of the cell index (CI) difference from the rolling average over 20 data points (10 proceeding and preceding CI values—5 min total). B) 50% inhibitory concentration (IC_50_) curves over time. Error bars represent 95% confidence intervals of nonlinear curve fit. The curves were marginally shifted on the x-axis to aid viewing. These figures represent the data from three independent studies.

Of the four compounds tested, (3*R*,6*R*)-linalool oxide acetate (**1**), luteolin (**3**) and luteolin-7-*O*-β-D-glucopyranoside (**4**) showed anti-schistosome dose-dependent anti-schistosome effects (Fig 2A). At the highest concentration tested (1 mg/mL) all three compounds killed flukes within 1–12 hr. Lower drug concentrations, however, took longer to kill flukes reflected by higher motility index values (Fig 2A).

When the IC_50_ values of each compound were averaged or combined (calculated for the dose concentrations of 0.1–1000 μg/mL) for each time point (1 hr, 6 hr and 12 hr), luteolin (**3**) and luteolin-7-*O*-β-D-glucopyranoside (**4**) appeared to be fast acting on worms as their IC_50_ values did not change significantly between the initial and final 12 hr dosing time points (Fig 2B). On the other hand, (3*R*,6*R*)-linalool oxide acetate (**1**) was slow-acting, showing two-fold decreases in IC_50_ at each 6 hr time point. Of the compounds assessed, luteolin (**3**) exhibited significantly better trematocidal activity at both 6 hr and 12 hr time points with IC_50_ values of 4.6 μg/mL and 5.8 μg/mL, respectively.

### Effects of compounds against schistosomula of *S*. *mansoni*

**Fig 3:**
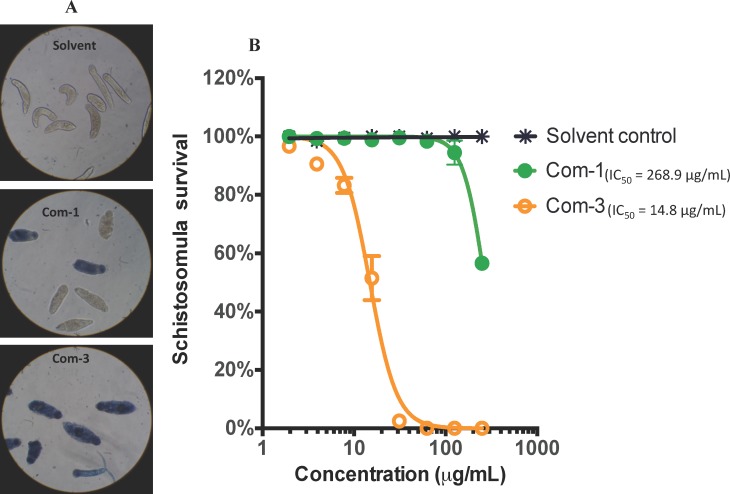
Effect of compounds on the survival of schistosomula. A) Images (20x) of wells containing schistosomula that were treated with linalool oxide acetate (**1**) and luteolin (**3**) (abbreviated as Com-1 and Com-3) at the maximum concentration tested of 250 μg/mL and stained with trypan blue. Deep blue staining signifies a dead parasite. B) The effect of (3*R*,6*R*)-linalool oxide acetate (**1**) and luteolin (**3**) (abbreviated as Com-1 and Com-3) on schistosomula mortality observed at two-fold dilutions (250–1.9 μg/mL). The data was generated from triplicate samples obtained from two independent studies.

The compounds that were active against adult *S*. *mansoni* were also tested against the intra-mammalian larval stage, the schistosomulum. Monitoring of schistosomula survival in the presence of different drug concentration using Trypan blue exclusion showed that luteolin (**3**) started to show lethal effects at the lowest dilution of 3.91 μg/mL and achieved 98–100% killing at the dilution of 31.3 μg/mL. (3*R*,6*R*)-linalool oxide acetate (**1**) however started to show lethal effects at a concentration of 125 μg/mL and only achieved about 43% killing at the maximum dose tested of 250 μg/mL. Schistosomula treated with 1% DMSO alone (solvent control) had 100% survival as measured by Trypan blue exclusion. A dose response curve of schistosomula survival after treatment with luteolin (**3**) revealed an IC_50_ of 13.3 μg/ml (Fig 3B).

### Effects of luteolin (3) on the morphology of adult *S*. *mansoni*

**Fig 4:**
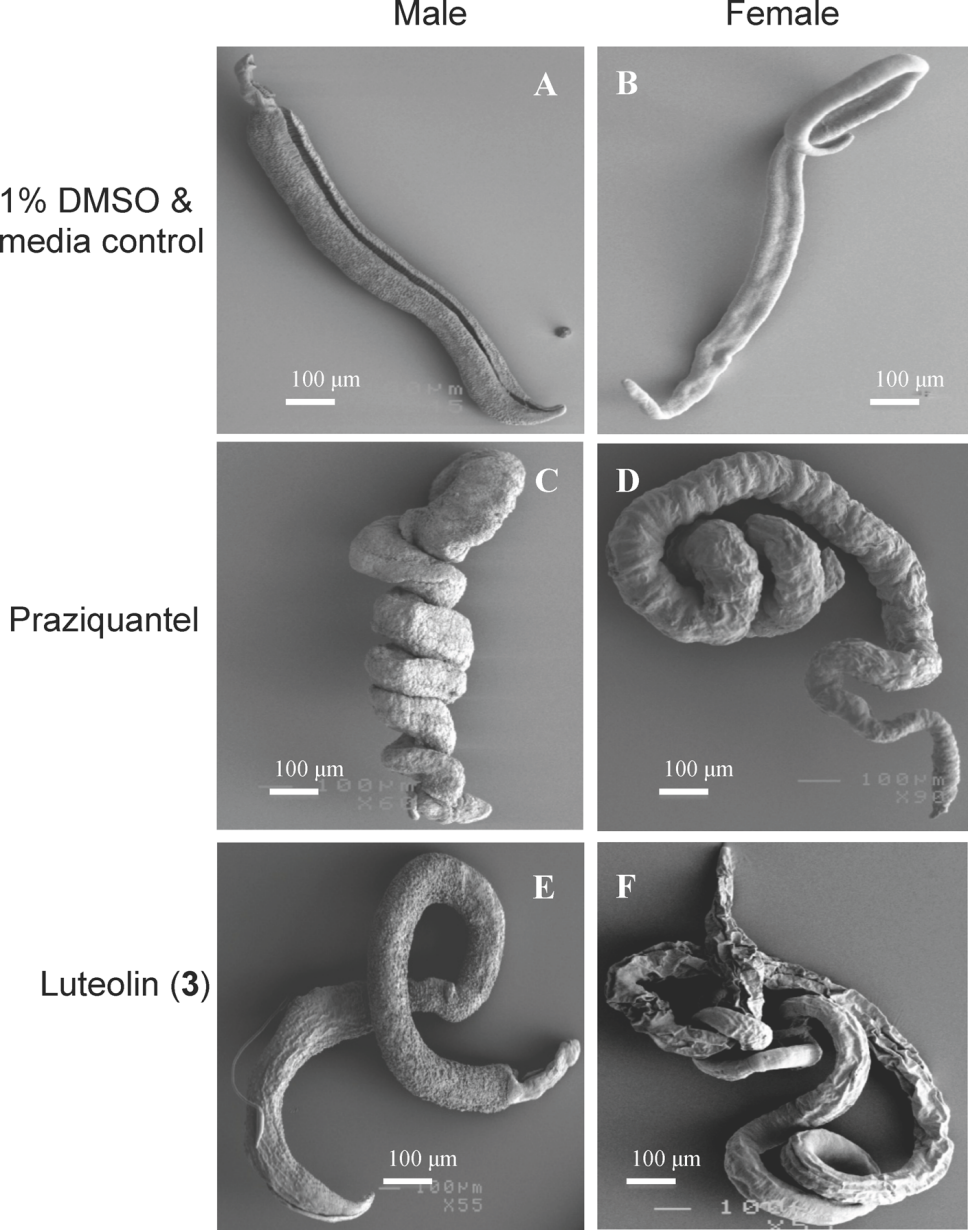
Effects of luteolin (3) and praziquantel on the morphology of adult *S*. *mansoni* at 4–20 μg/mL concentration visualized using scanning electron microscopy. A, C and E show male flukes. B, D and F depict female flukes. (A, B) Flukes in DMSO/media control. (C, D) Flukes treated with praziquantel (tightly coiled). (E, F) Flukes treated with luteolin (**3**) (moderately coiled). Three separate wells (with 3–5 adult flukes per well) were treated with different doses of test samples as above and each of them was examined under SEM.

Luteolin (**3**) demonstrated the best anthelmintic activity when motility was assessed using xWORM. The effect of this compound and praziquantel on adult fluke morphology at 4–20 μg/mL concentration, with particular emphasis on the tegument, was assessed by SEM. Adult flukes treated with praziquantel adopted a tightly coiled appearance due to contraction. Both male and female flukes treated with luteolin (**3**) were contracted and coiled compared to control flukes cultured in 1% DMSO in media, but not as tightly coiled as praziquantel-treated parasites (Fig 4A–4F).

**Fig 5:**
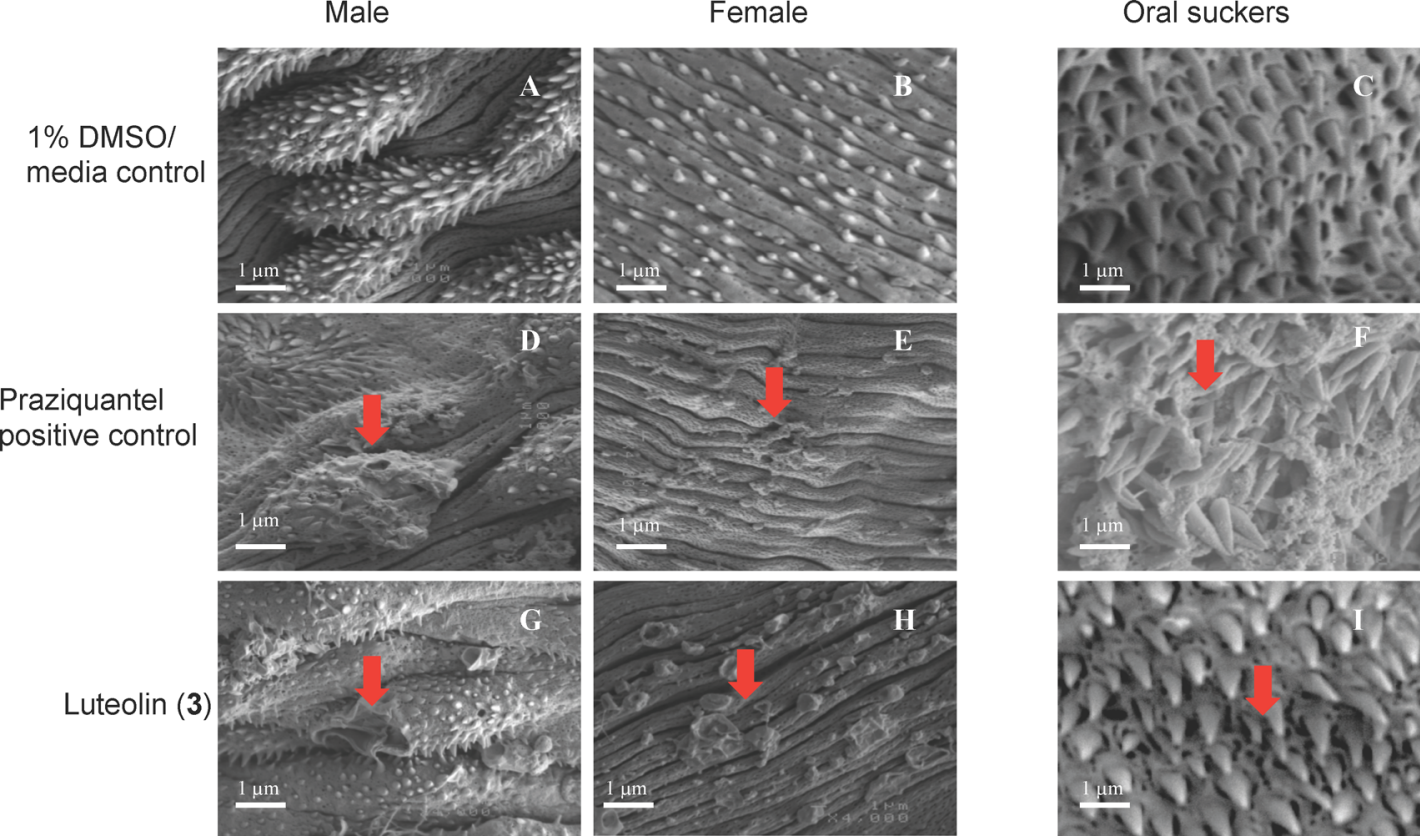
Scanning electron micrographs of *S*. *mansoni* adult fluke teguments after treatment with 4 μg/mL concentration of luteolin (3) and praziquantel. A, B and C represent flukes treated with solvent (1% DMSO in culture media). A) Male tegument with well defined tubercles. B) Female tegument with clearly defined grooves and sensory papillae. C) Oral sucker with well formed pits containing spines. D, E and F represent flukes treated with praziquantel. D) Male tegument with damaged tubercles and spines. E) Female tegument with damaged papillae and swollen grooves. F) Oral suckers with eroded pits and detached spines. G, H and I represent flukes treated with luteolin (**3**). G) Male tegument with destroyed tubercles and holes. H) Female tegument with burst sensory papillae and holes. I) Oral suckers with partially eroded pits. Arrows in panels highlight treatment effects described in results text. All images at ×4000 magnification and 1 μm are scale bars shown.

Observation of fluke teguments by SEM under high magnification revealed that the tegument of DMSO-treated flukes displayed numerous tubercles, with well-formed spines in the males (Fig 5A) and clearly defined surface grooves with sensory papillae in females (Fig 5B), and oral suckers with clearly defined pits containing sharp spines (Fig 5C). Flukes treated with praziquantel (Fig 5D–5F) and luteolin (**3**) (Fig 5G–5I) exhibited severe morphological alterations of the tegument.

At the lowest concentration tested of 4 μg/mL we observed clear tegumental damage induced by luteolin (**3**) (Fig 5G–5I), similar to that induced by praziquantel (Fig 5D–5F). While male flukes (Fig 5G) suffered partial loss of pits and their encased spines, female flukes (Fig 5E) showed surface erosion, constriction of grooves, bursting of small sensory papillae and formation of holes on the tegument. At higher concentrations of 20–1000 μg/mL, luteolin (**3**) and praziquantel completely destroyed the body surfaces and exhibited erosion of tubercles, oral and ventral suckers (S1 Fig).

### Nematocidal activities of compounds against adult *T*. *muris*

**Fig 6:**
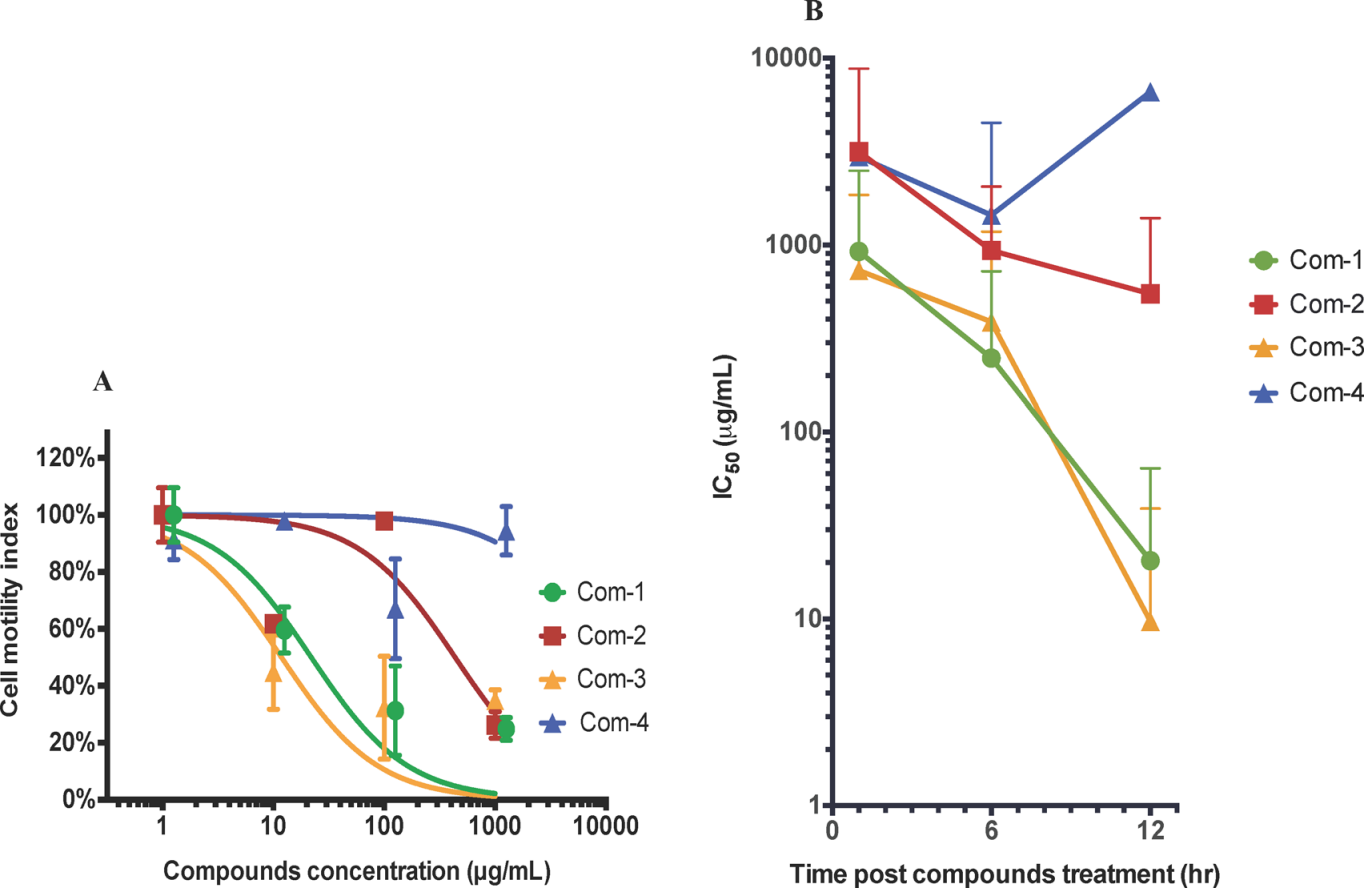
Anti-*Trichuris* activity of compounds (1–4) on adult *T*. *muris*. A) Motility index dose response curve of worms at the 12 hr time point when treated with (3*R*,6*R*)-linalool oxide acetate (**1**), (*E*)-spiroether (**2**), luteolin (**3**) and luteolin-7-*O*-β-D-glucopyranoside (**4**) (abbreviated as Com-1, Com-2, Com-3 and Com-4) at different concentrations. Motility index was calculated as the standard deviation (SD) over 800 data points (i.e. 4 readings per min for 200 min) of the cell index (CI) difference from the rolling average over 20 data points (10 proceeding and preceding CI values—5 min total). B) Combined IC_50_ values of these four compounds calculated for three different doses at 1 hr, 6 hr, and 12 hr time points. Error bars represent 95% confidence intervals of nonlinear curve fit. The curves were marginally shifted on the x-axis to aid viewing. These figures represent the data from three independent studies.

Prior to testing the nematocidal effects of (3*R*,6*R*)-linalool oxide acetate (**1**), (*E*)-spiroether (**2**), luteolin (**3**) and luteolin-7-*O*-β-D-glucopyranoside (**4**), we standardized the culturing conditions of adult *T*. *muris* for the xWORM assay. E-plate wells containing four adult worms (both males and females) yielded optimal motility signals (S2 Fig), and was the condition selected for subsequent anthelmintic screening of the test compounds. (3*R*,6*R*)-linalool oxide acetate (**1**) and luteolin (**3**) showed the best anti-*Trichuris* activity with IC_50_ values of 20.4 μg/mL and 9.7 μg/mL, respectively, calculated on cell motility index at the 12 hr time point (Fig 6A).

The IC_50_ values of the four compounds tested here were obtained using xWORM and calculated at 1 hr, 6 hr and 12 hr time points (Fig 6B). Luteolin (**3**) was the most efficacious drug in terms of reduced motility of *T*. *muris*, exhibiting the lowest or equally low IC_50_ values at all time points and a final 12 hr value of 9.7 μg/mL.

### Effects of luteolin (3) on the morphology of adult *T*. *muris*

**Fig 7:**
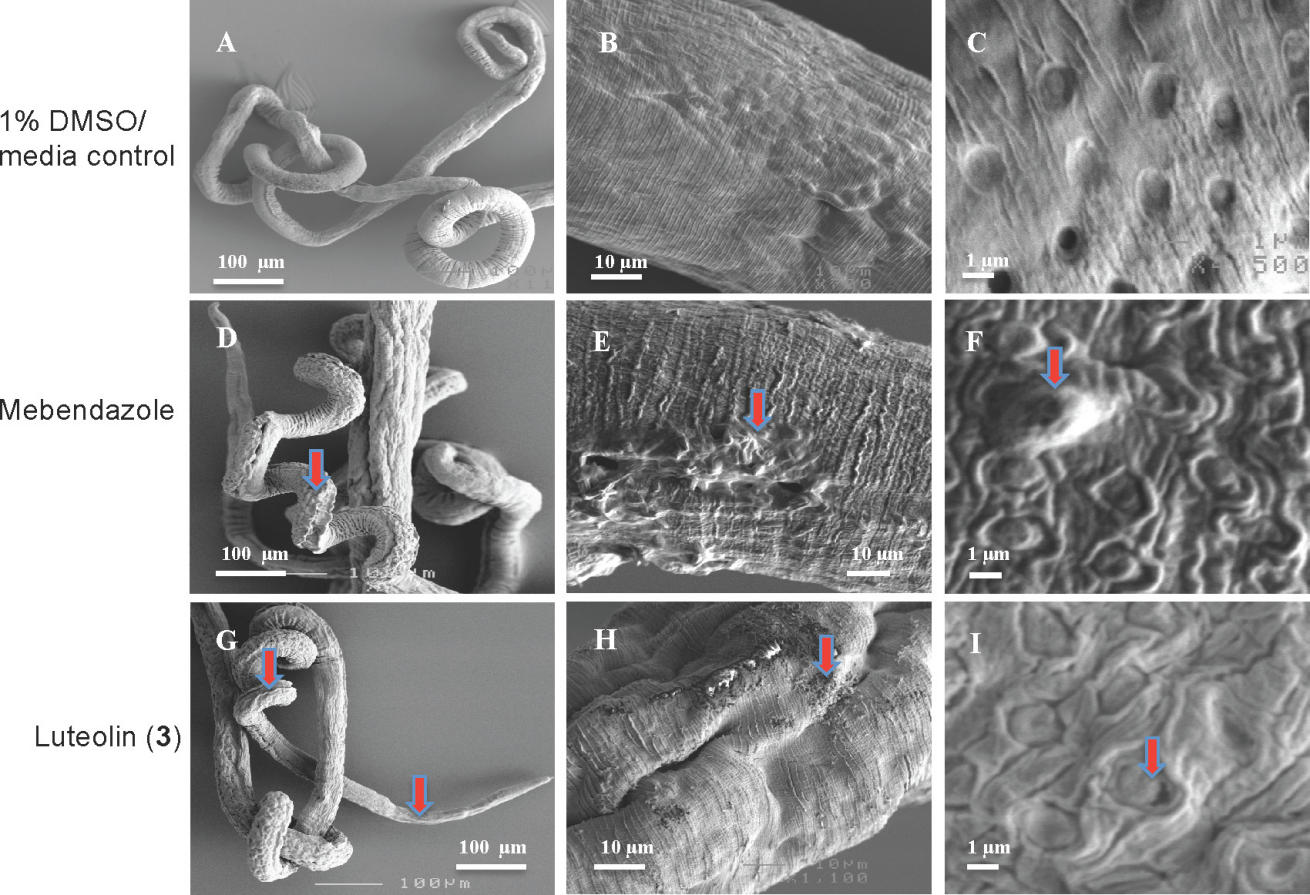
Effects of luteolin (3) and mebendazole on the cuticle of adult *T*. *muris*. A) Normal appearance of control worms cultured in 1% DMSO/media (x100) and at higher magnification focusing on the cuticle (B) and bacillary gland (C), respectively. D) Shrinkage of the anterior regions of worms after treatment with mebendazole and damage to the cuticle (E) and bacillary glands (F) were observed at higher magnification. G) Shrinkage of the anterior regions of worms after treatment with luteolin (**3**) and damage to the cuticle (H) and swelling of bacillary glands (I) observed at higher magnification. Red arrows in all panels highlight treatment damage described in results text.

Based on the efficacy of luteolin (**3**) at reducing *Trichuris* motility, we examined the morphological changes in the cuticle induced by this compound using the SEM protocols described by Stepek et al [30] and Tritten et al [31] specific to *T*. *muris*. Live adult *T*. *muris* (mixed sexes) were treated for 48 hr with luteolin (**3**) or mebendazole at dose concentrations of 200–1000 μg/mL. Morphological changes were observed towards the anterior end of the worms in the form of partially damaged bacillary band/glands and adjacent cuticle. The bacillary band is a specialized row of longitudinal cells of some nematodes consisting of glandular and non-glandular cells. These bands host the glands. Worms treated with DMSO/media (solvent control) alone had a moderately coiled appearance with a smooth cuticle displaying knitted parallel segmental joins and minimal shrinkage of bands (Fig 7A–7C) in comparison to the groups treated with mebendazole (Fig 7D–7F) and luteolin (**3**) (Fig 7G–7I). At higher magnification we observed that the luteolin-treated worms exhibited blister-like formations on the surface of the cuticle, swelling and loosening of cuticle seams/grooves near the bacillary glands (Fig 7I). These morphological changes were similar to that of the mebendazole-treated worms (Fig 7F).

### Anti-*Trichuris* effect of luteolin (3) in mice infected with *T*. *muris*

**Fig 8:**
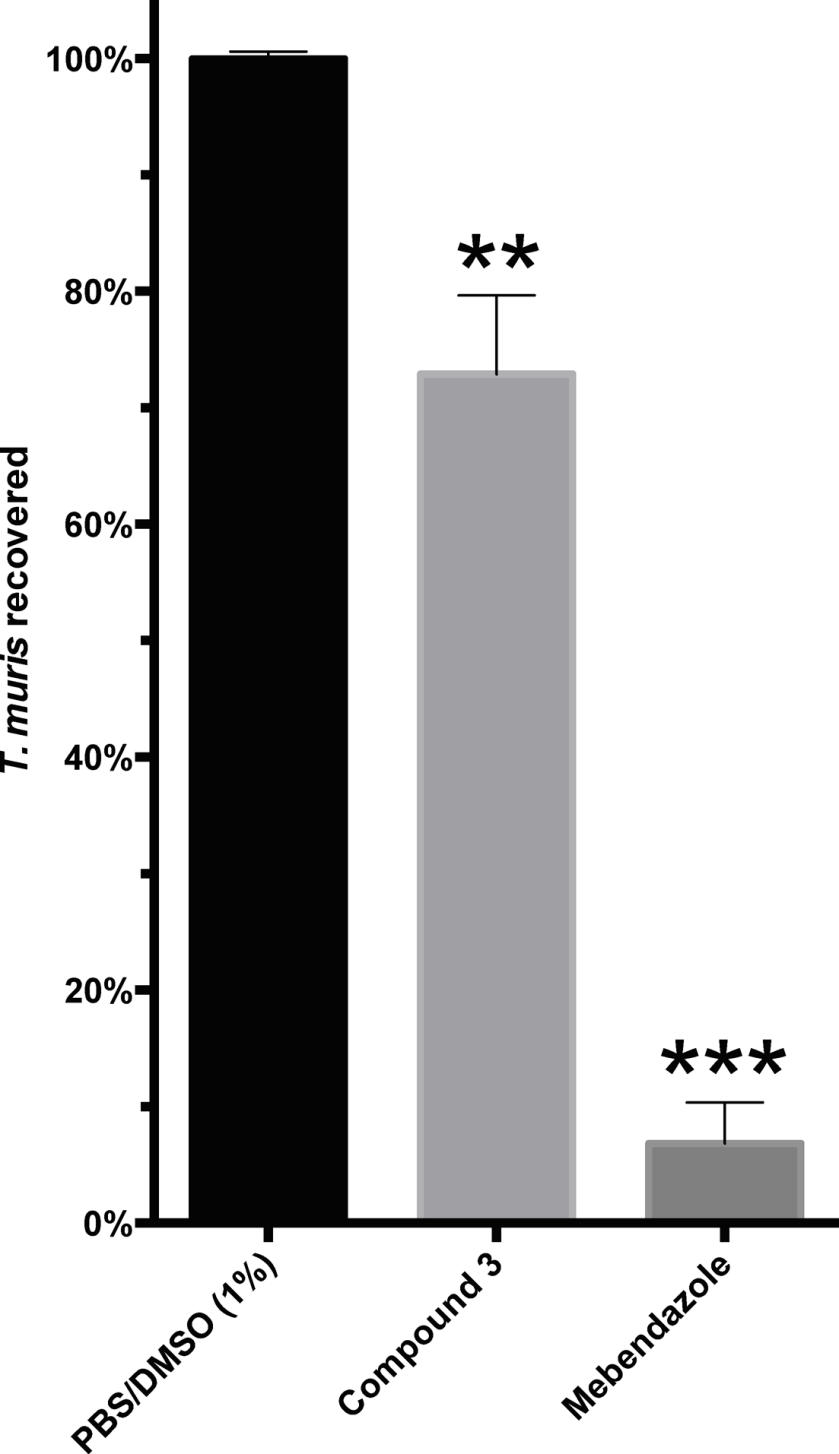
Effects of luteolin (3) and mebendazole on *T*. *muris* burden in mice. The graph represents the percentage of worms recovered from the STAT6^*-1-*^ mice five days after a single oral dose of luteolin (**3**) (27.1% reduction with *p*-value of 0.0087) and the positive control drug, mebendazole (93.1% reduction with *p*-value of 0.0005). The *p*-values were determined by one-way ANOVA Holm-Sidak’s multiple comparisons test. Each experimental group consisted of nine mice.

Based on the significant *in vitro* nematocidal activity demonstrated by luteolin (**3**), this compound was further assessed for its anti-*Trichuris* effect *in vivo* using a mouse model of *T*. *muris* infection [29–31]. Four weeks post-infection with *T*. *muris* eggs, mice were administered with a single 100 mg/kg oral dose of luteolin (**3**). When mice were sacrificed 5 days later we observed that a single treatment of this compound resulted in a 27.6% reduction in worm burdens (249 worms); luteolin (**3**)–treated mice = 651 worms; DMSO-treated mice = 900 worms; P = 0.0087) (Fig 8). The positive control drug, mebendazole, reduced worm burdens by 93.1% (mebendazole treated mice = 50 worms). The activity of luteolin (**3**) against *T*. *muris*, despite being significantly better than the untreated control group, was markedly weaker than mebendazole in the mouse model. Mice treated with luteolin (**3**) did not show signs of toxicity at any of the concentrations tested.

## Discussion

Globally, helminth infections caused by roundworms (nematodes) and flatworms (platyhelminths) comprise the largest group of NTDs [2]. Schistosomiasis (caused by the platyhelminth blood flukes) and trichuriasis (caused by the nematode whipworms) affect about 240 million and 800 million people, respectively [3–4]. While schistosomiasis is transmitted through infected snails and water, trichuriasis is transmitted through soil and is therefore referred to as a soil-transmitted helminth infections (STHI). The STHI are among the most common infections worldwide and affect the poorest and most deprived communities where sanitation is poor. Sole reliance on praziquantel for schistosomiasis, and only a very small number of drugs (some with poor cure rates) for STHI, has precipitated the need for new anthelmintic drugs to treat parasites that infect both humans and animals [7]. In this context, medicinal plants present a viable source of novel anthelmintic compounds. Indeed, many anti-parasitic drugs including quinine, chloroquine, artemisinin and atovaquone were originally purified from plants. Our initial study, involving the crude CHCl_3_ extract of *A*. *nubigena* (syn. *Tanacetum nubigenum* DC.) and its compounds luteolin and luteolin-7-*O-β-*D-glucopyranoside, showed significant antiparasitic activities against the multidrug resistant K1CB1 strain and chloroquine sensitive TM4/8.2 strain of *Plasmodium falciparum* [16, 21]. Interestingly, this plant and its close relatives have been used in the ethnomedicines for treating arrays of disorders including wound, bleeding and worm infection [16, 21–23].

In this study, we have demonstrated that compounds linalool oxide acetate (**1**) and luteolin (**3**) had significant trematocidal activity against *S*. *mansoni* and nematocidal activity against *T*. *muris*. These compounds are simple small secondary plant metabolites belonging to the terpenes and flavonoids. Luteolin (**3**) was the most active compound against both parasites with IC_50_ values of 5.8 μg/mL against *S*. *mansoni* and 9.7 μg/mL against *T*. *muris* calculated at the 12 hr time point. It also effectively killed schistosomula with an IC_50_ value of 13.3 μg/mL. Intriguingly, this same compound has been shown to have significant anti-malarial activity against *P*. *falciparum* TM4/8.2 (chloroquine-antifolate sensitive strain) and K1CB1 (multidrug resistant strain) [21]. New anti-parasitic drugs require excellent safety and therapeutic profiles, should exhibit broad spectrum activity against different types of infections, and also display significant activity against different developmental stages of parasites. Current anthelmintic drugs are generally effective at treating single stages of target helminths. For example, praziquantel is effective against adult stage schistosomes but not schistosomula/cercariae. Therefore, finding a broad-spectrum drug that could treat multiple diseases or multiple life stages is desirable when treating large populations in resource-poor settings. Luteolin (**3**) met these criteria in that it has anti-malarial [21], anti-fluke and anti-whipworm properties. In addition, this compound was effective in killing schistosomula, the stage of S. *mansoni* that is unaffected by praziquantel.

While our findings do not specifically address the mechanism of action of these anthelmintic compounds, we showed that luteolin (**3**) is capable of damaging the outer surface membranes of the parasites–the fluke tegument and the nematode cuticle and associated glandular structures, and worms adopted a coiled state of paralysis. Previous studies on *S*. *mansoni* have demonstrated that the tegument plays essential roles in many processes at the host-parasite interface, and numerous molecular pathways that are represented at the host-parasite boundary are anti-parasitic drug targets [5, 32]. SEM has been used to demonstrate the mechanisms of many anti-parasitic drugs, including oxamniquine, praziquantel, mefloquine, mebendazole and artemisinin [20, 32–39]. Schistosomes treated with these drugs displayed vacuolization or bubble-like-lesions, surface erosion, destruction of tubercles and tissues, loss of sensory papillae, and pore formation leading to death. SEM has also been used to reveal cuticular damage in nematodes, particularly for the benzimidazole class of drugs [40–41]. Stepek et al [30] and Tritten et al [31] first used the plant extracts and nitazoxanide to demonstrate the mechanisms of the antiparasitic action against *T*. *muris*. These same morphological changes were observed in adult *S*. *mansoni* and *T*. *muris* that were treated with luteolin (**3**), suggesting that the mechanism of action is similar to at least some of the existing anthelmintic drugs. For *T*. *muris*, luteolin (**3**) and mebendazole mainly affected the anterior bacillary band and surrounding glands. The anterior ventrolateral face of the worm contain the glandular pores, bacillary band sensory cells and glands, and the stichosome cells which helps in the formation of the syncytial feeding site in the host, and plays an important role in the excretion of digestive enzymes, pre-digestion and nutrient uptake in *Trichuris* [42–45].

Luteolin (**3**), with a Log*P* value of 2.6, meets the Lipinski rule of 5 criterion for drug-likeness [46]. Generally, compounds with Log*P* values in the range of 2–3 are more likely to diffuse/permeate through the cell membrane of an organism, and therefore enabling them to interact with target receptors. There was no structural similarity between our active compounds and the currently used anthelmintic drugs, which suggest that compounds that damage the worm surface do not necessarily have similar structural scaffolds. It should be noted that our *in vivo* assessment of luteolin (**3**) against *T*. *muris* infection at a single oral dosing of 100 mg/kg, despite being significantly (27.6%) better than the untreated control group, was markedly weaker than mebendazole (93.1%) in reducing the worm burden in mice. Moreover, the mice showed no signs of ill health, suggesting that luteolin (**3**) is not overtly toxic and allows future studies to explore the efficacy of multiple treatments. We did not assess *in vivo* efficacy of luteolin (**3**) against *S*. *mansoni* in the mouse model. Future work will entail synthesis of luteolin and its derivatised compounds in a thorough *in vivo* assessment of efficacy in mouse models of schistosomiasis and trichuriasis, as well as other soil-transmitted helminth infections.

## Supporting Information

S1 Fig SEM images of damaged tubercles, oral and ventral suckers (anterior parts) of *S*. *mansoni* (male) when treated with luteolin (3) and praziquantel at a dose concentrations of 20–1000 μg/mL. Praziquantel treated groups (A and C). Luteolin treated groups (C and D).
(TIFF)

S2 Fig Motility signals of *T*. *muris* (mixed male and female) obtained by xCELLigence with 2–16 worms per well. A) depicts the Motility Index of 2–16 worms per well over 12 hours. B) depicts the average blanked Motility Index of 2–16 worms per well over 12 hours with standard deviation error bars (generated from data shown in A). Four worms per well was determined as optimal as the motility index signal was the most stable over time, with lowest variation.
(TIF)
