# Supplementary figures and images for: Compounds Derived from the Bhutanese Daisy, Ajania nubigena, Demonstrate Dual Anthelmintic Activity against Schistosoma mansoni and Trichuris muris
Source: PLoS Negl Trop Dis. 2016 Aug 4;10(8):e0004908. doi: 10.1371/journal.pntd.0004908 (PMC4973903; doi:10.1371/journal.pntd.0004908)

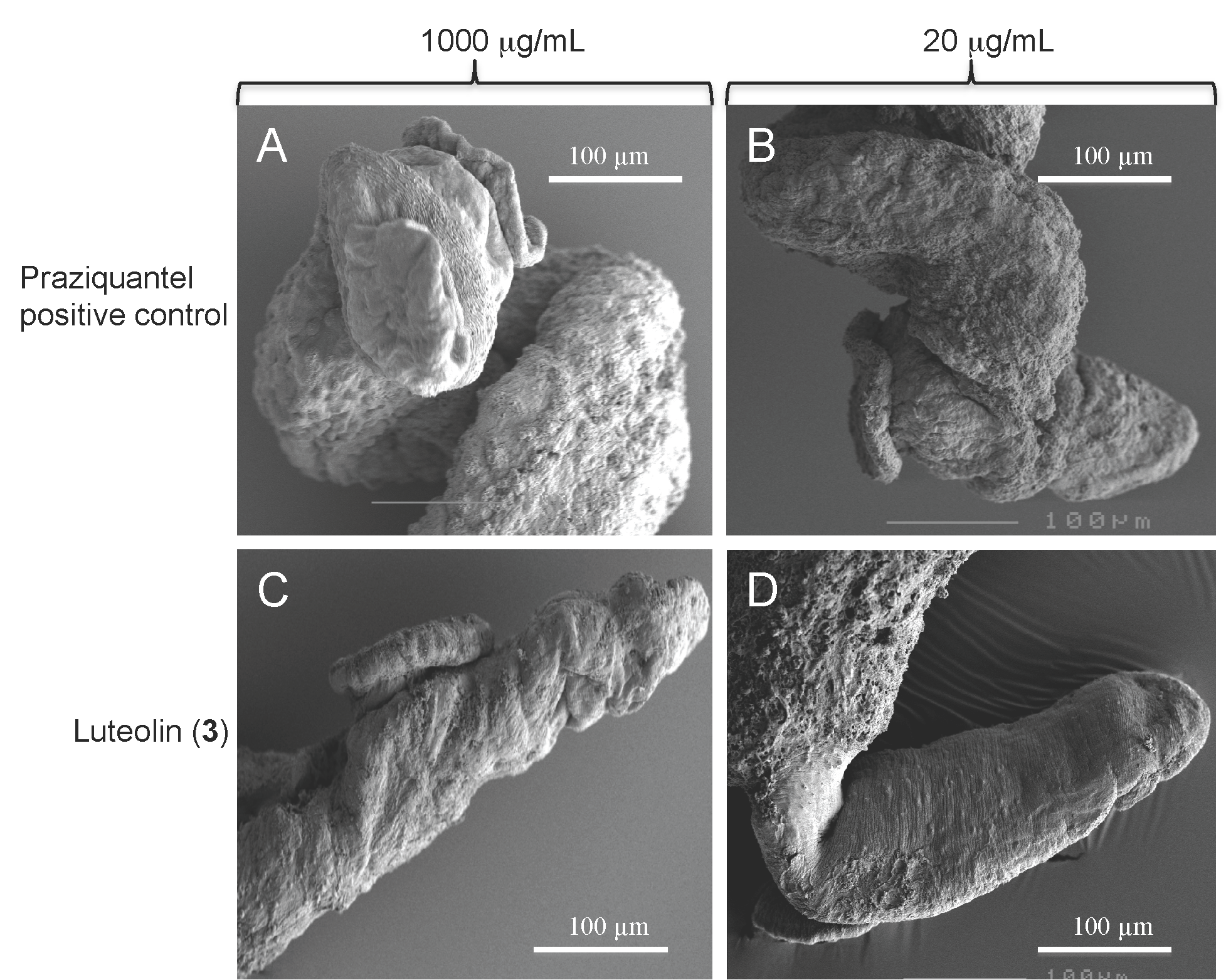

Supplement: S1 Fig — Praziquantel treated groups (A and C). Luteolin treated groups (C and D). (TIFF) [file pntd.0004908.s001.tiff]

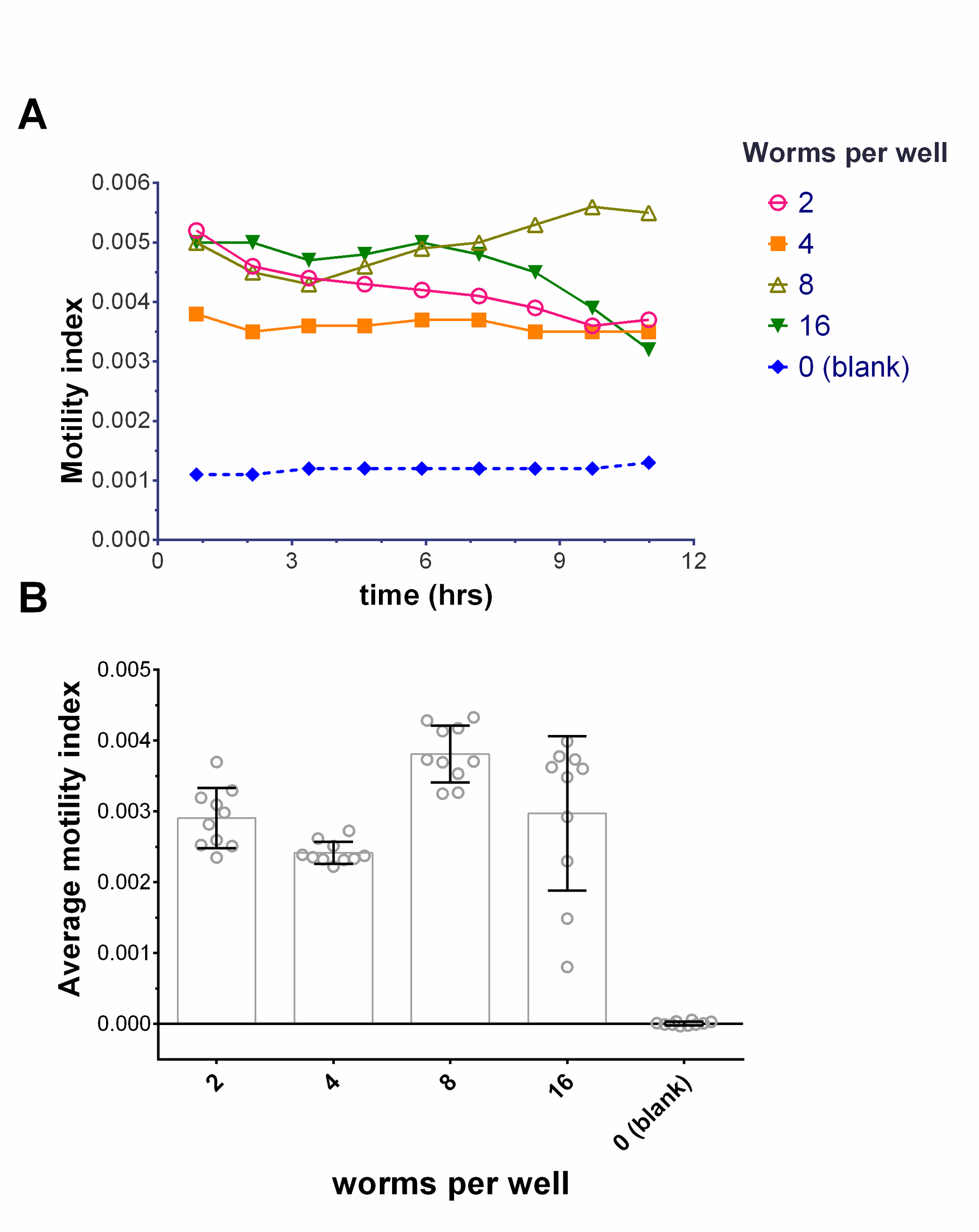

Supplement: S2 Fig — A) depicts the Motility Index of 2–16 worms per well over 12 hours. B) depicts the average blanked Motility Index of 2–16 worms per well over 12 hours with standard deviation error bars (generated from data shown in A). Four worms per well was determined as optimal as the motility index signal was the most stable over time, with lowest variation. (TIF) [file pntd.0004908.s002.tif]
